# Study on the negative effect of internal-control willingness on enterprise risk-taking

**DOI:** 10.3389/fpsyg.2022.894087

**Published:** 2022-07-22

**Authors:** Lijun Chen, Yanxi Li, Bin Liu

**Affiliations:** ^1^School of Economics and Management, Dalian University of Technology, Dalian, China; ^2^School of Management, Hainan University, Haikou, China

**Keywords:** internal-control willingness, internal-control level, enterprise risk-taking, text analyses, machine learning

## Abstract

In the traditional cognition, the factors that affect the level of internal control are usually based on the objective factors such as corporate characteristics, financial status, and governance structure. However, the internal control defects of many famous companies expose the phenomenon of subjective manipulation, and this leads us to focus on the subjective factor of internal control, which we call internal-control willingness. We define “internal-control willingness” as the degrees of subjective initiative of the internal-control construction and execution activities. Additionally, we propose a method for measuring internal-control willingness, using text analysis and machine learning. Then, we examine the impact of internal-control willingness on enterprise risk-taking, through the internal-control, financial, and market data of China A-share main board enterprises in 2011–2018. The study found that (1) internal-control willingness has a significant positive impact on internal-control level, which can fairly achieve the measurement of internal-control subjective initiative. (2) It confirms that internal-control willingness lowers corporate risk-taking. (3) Further research finds that state-owned enterprises strengthen internal-control willingness and their risk-taking level is significantly lower than that of non-state-owned enterprises. This paper suggests that the regulatory authorities actively urge the board of directors to strengthen internal-control willingness.

## Introduction

In the traditional cognition, the factors that affect the level of internal control are usually based on the objective factors such as corporate characteristics, financial status, and governance structure (Doyle et al., 2007; Ashbaugh-Skaife et al., 2009). However, there are also phenomena that cannot be explained by objective factors. For example, in enterprise practice, the internal control defects of Enron and other companies expose the phenomenon of subjective manipulation, resulting in bankruptcy (Deakin and Konzelmann, 2004). According to the Positive Accounting Theory, the growing risk of bankruptcy is associated with the phenomenon of earning management (Durana et al., 2021). Earning management is one of the behaviors that is subjectively divorced from the internal control of an enterprise. It shows the important impact of the subjective will of internal control on the level of enterprise risk. For example, although the accounting standards and regulations governing all United States companies are the same, Enron has committed extremely serious fraud. Therefore, “Enron” must have different degrees of internal-control willingness (ICW) from other companies, which leads to its committing these fraudulent acts.

*Willingness*, as a term in psychology, is mainly used in the field of economics to measure the degree of subjective will of actors, such as “willing to pay” and “willing to accept,” “willing to participate,” “consumer willingness,” and so on (Füller et al., 2010; Phelps et al., 2013; Jong et al., 2022). At the same time, the subjective initiative of internal-control construction activities is a verifiable and objectively existing natural phenomenon of applied psychology (Liu and Li, 2021; Peng and Yan, 2021). Therefore, we define “internal-control willingness” as the degrees of subjective initiative of the internal-control construction and execution activities.

Although internal-control willingness is an important part of internal-control system, there is a lack of research on this subject. The main obstacle is that the willingness of internal control is subjective in nature, so it is unobservable. Therefore, the attention will be focused on the following questions: (A) How to identify, confirm, and measure internal-control subjective factor (willingness) reliably? (B) What are the economic consequences, based upon the difference of internal-control willingness on corporate risk level? Can those enterprises with positive internal-control subjective willingness lower their current level of risk-taking in construction and execution of internal-control system? All of the questions mentioned above will be answered in the following analysis.

The contributions of this paper are as follows. The first contribution is the introduction of subjective factor “internal-control willingness” into the research field of internal control. In previous researches, scholars have focused on the objective factors, such as corporate characteristics, financial status, and governance structure (Doyle et al., 2007; Ashbaugh-Skaife et al., 2009). Additionally, our research focuses on the influence of subjective factors and introduces a methodology that quantifies “internal-control willingness.” The second contribution is to discover the negative effect of internal-control willingness on enterprise risk-taking and the heterogeneity of the nature of property rights. In previous researches, scholars have focused on the overall internal-control level that has a significant negative impact on the level of enterprise risk-taking (Bargeron et al., 2010; Chen et al., 2020; Baugh et al., 2021). Additionally, our research discovers the subjective factor of internal control on the level of enterprise risk-taking. Moreover, we also discover that state-owned enterprises to strengthen internal-control willingness, their risk-taking level is significantly lower than that of non-state-owned enterprises.

## Research hypotheses

The United States COSO Reports (1992, 2004, 2013, 2017), the SOX Act (2002), and the Corporate Internal Control Basic Standards in China (2008) all stated that the goals of the internal-control act include: “reasonably ensure the legality and compliance of business operation and management,” “ensure asset security,” “financial reports and related information are true and complete,” “improve operating efficiency and effectiveness,” and “promote the realization of development strategies for enterprises” (Chen et al., 2017; Chalmers et al., 2019). The realization of the above goals depends on the dominant position of board of directors in internal-control construction and also on the collective “willingness” of governance, management, and all employees. Therefore, there are the following logical chains among internal-control goals, subjective willingness, and final result of internal control (internal-control quality). Logically, internal-control willingness and internal-control quality have consistent goals.

According to the theory of “Positive Organizational Behavior” (Luthans, 2002), “personal positive psychology in organization → organizational tendency and behavior → realization of organizational (performance) goals” is a complete logical framework. We place the research framework above in the research field of internal control, and we can get a new logical framework like this, that is “internal-control (positive) willingness → internal-control tendency and behavior → internal-control results (internal-control quality).” We put aside the intermediary link. Logically speaking, internal-control willingness is a component of internal-control results (internal-control quality). Thus, we first need to clarify the relationship between internal-control willingness and internal-control level (internal-control quality). We propose a basic hypothesis:

**Hypothesis 1.** Internal-control willingness has a positive impact on internal-control level.

The United States COSO Reports, the SOX Act, and the Corporate Internal Control Basic Standards in China all clarify that the formulation goal of internal-control acts is to strengthen and standardize internal control, to improve business management level and risk prevention capabilities, and to safeguard market order and public interests. Among them, the goal of improving management level and ability of risk prevention is consistent in purpose with the subjective willingness to actively construct and execute internal-control system. The phenomenon is particularly evident in the financial industry. Additionally, empirical evidence suggested that financial institutions that complied with the Federal Deposit Insurance Corporation Improvement Act (FDICIA) and Internal-control Guidelines for Commercial Banks in China could significantly reduce the risk of financial crisis and distress and then lower corporate risk-taking level (Jin et al., 2013; Chen et al., 2016; Li et al., 2021). Logically, internal-control willingness is negatively correlated with the current level of corporate risk-taking.

At the same time, previous studies have shown that high-quality internal-control level had a significantly negative impact on corporate risk-taking level (Bargeron et al., 2010; Chen et al., 2020; Baugh et al., 2021). Furthermore, internal-control willingness is an important precipitation component of the overall internal-control level. Therefore, from a logical point of view, it can be expected that internal-control willingness has a negative impact on corporate risk-taking. Based on this, this paper proposes the second hypothesis:

**Hypothesis 2.** Internal-control willingness could lower corporate risk-taking.

## Research design

### Confirmation and measurement of internal-control willingness

*Willingness*, as a term in psychology, is mainly used in the field of economics to measure the degree of subjective will of actors (Liu and Li, 2021). The traditional “willingness” research usually adopts the method of questionnaire. However, the questionnaire is easily affected by the subjective factors of the respondents, and its reliability and validity are also questioned (Coursey et al., 1987; Jong et al., 2022). Therefore, we use text analysis and machine learning to measure internal-control willingness, which can more objectively mine the managerial public information in the annual internal-control report, and avoid the limitations of the questionnaire.

#### The confirmation starting point of internal-control willingness and the text source of “key characteristics”

Internal-control willingness can be interpreted as the degree of subjective initiative in the construction and implementation of internal control. This study takes the enterprise samples of “voluntary pilot units of internal control” of Shanghai and Shenzhen stock exchanges as the starting point of Python text analysis, because these “voluntary” enterprises that take the lead in the construction of internal control are most likely to carry out the construction and implementation of enterprise internal control with “positive will.” The information is disclosed through the “enterprise internal-control self-evaluation report” issued by the board of directors. Therefore, we are most likely to obtain the “key features” and statistical probability data of the “positive willingness” of internal control for Python to carry out subsequent text feature analysis and machine learning. Specifically, in the sample interval of this study from 2011 to 2018, we found 188 enterprises that may have the above “key characteristics” of annual internal-control evaluation reports.

#### Measurement method of machine learning and internal-control willingness based on Python

We conducted lexical text analysis on 188 enterprises of annual internal-control evaluation reports in the above “voluntary pilot units of internal control.” We finally got 45 groups of “Keywords” of “positive willingness” to be the “key characteristics” of “positive willingness” of internal control. These “key characteristics” are selected from high to low according to the statistical probability of vocabulary occurrence. It should be noted that other “Keywords” with lower ranking are no longer significant in subsequent machine learning applications, so they are not selected. We use these “key characteristics” to form a “word bag” for Python to carry out subsequent machine learning.

Based on Python’s Scikit-Learn library, we use the “key characteristics” as the feature variable and use its probability “word frequency” to construct the word frequency matrix for text vectorization. Then, the training algorithm model is constructed based on the KMeans algorithm in cluster analysis, and the contents of each annual internal-control evaluation report of all samples are clustered. According to the “prediction” results of clustering, the internal-control willingness shown in each report is distinguished and confirmed as “positive willingness” or “other willingness.” For the measurement of internal-control willingness, we adopt two classifications based on the “prediction” results of Python machine learning: one is the enterprise with positive will, and the set value is “1”; the other is the sample of other enterprises, and the set value is “0.”

### Measurement of enterprise risk-taking level

As for the measurement of enterprise risk-taking level, previous studies mainly adopt two kinds of methods: first, using the volatility index of stock return in the capital market (Coles et al., 2006); second, using the volatility indicators of earnings or earnings, such as ROA* (John et al., 2008; Faccio et al., 2011). This paper uses the latter method for reference and measures the *RiskT* of the enterprise based on the volatility of σ(ROA*). The measurement method is designed as follows:


(1) $$R\,i\,s\,k\,T_{\text{it}}=\sqrt{\frac{1}{T-1}\sum_{t=1}^{T}{\left(Ad\text{j}ROA{*}_{i,t-1}-\frac{1}{T}\sum_{t=1}^{T}Ad\text{j}ROA{*}_{i,t-1}\right)}^{2}}||T=3$$

(2) $$A\text{dj}ROA{*}_{\text{it}}=\frac{E\,B\,I\,T\,D\,A_{\text{it}}}{A_{\text{it}}}-\frac{1}{{\text{X}}_{\text{t}}}\sum_{K=1}^{\text{X}}\frac{{\text{EBITDA}}_{\text{kt}}}{{\text{A}}_{\text{kt}}}$$

wherein ROA* is the ratio of the profit before interest and tax (*EBITDA*) of enterprise *i* in year t to the total assets at the end of the year; AdjROA* refers to the value of enterprise *i* adjusted by the average value of ROA* of the industry in which the enterprise is located in t year. Its purpose is to eliminate the impact of industry factors on *ROA*. T refers to the number of statistical years of calculation σ(ROA*). Based on the employment characteristics of enterprise executives, this paper calculates *RiskT* with 3 years (t-1, t, t+1) as a cycle, to eliminate the influence of cyclical factors. *RiskT* refers to the final risk bearing level of enterprise *i* in year *t*; the bigger the *RiskT*, the higher the risk-taking level of the enterprise.

### Test model design

The regression model of this paper is based on the control factors confirmed by many scholars in the research field of influencing factors of internal-control level (Doyle et al., 2007; Ashbaugh-Skaife et al., 2009) and many scholars in the research field of enterprise risk-taking and internal control (John et al., 2008; Faccio et al., 2011) and introduces the internal-control willingness factor. Models (3) and (4) are the main test models for this study. The variable descriptions are detailed in Table 1.


(3) $$I\,C_{\text{it}}=\alpha_{0}+\alpha_{1}\,I\,C\,W_{\text{it}}+\alpha_{2}\,S\,{\text{ize}}_{i\,t}+\alpha_{3}\,L\,{\text{ev}}_{\text{it}}+\alpha_{4}\,B\,S_{\text{it}}+\alpha_{5}\,R\,O\,I\,D_{\text{it}}+\alpha_{6}\,E\,I_{\text{it}}+\alpha_{I\,n\,d}\,I\,n\,d_{i\,t}+\alpha_{Y\,e\,a\,r}\,Y\,e\,a\,r_{i\,t}+\varepsilon_{i\,t}$$

(4) $$R\,i\,s\,k\,T_{i\,t}=\beta_{0}+\beta_{1}\,I\,C\,W_{i\,t}+\beta_{2}\,S\,i\,z\,e_{\text{it}}+\beta_{3}\,E\,I_{i\,t}+\beta_{4}\,G\,{\text{rowth}}_{\text{it}}+\beta_{5}\,F\,A\,g\,e_{i\,t}+\beta_{6}\,H\,e\,r\,f_{i\,t}+\beta_{I\,n\,d}\,I\,n\,d_{i\,t}+\beta_{Y\,e\,a\,r}\,Y\,e\,a\,r_{i\,t}+\varepsilon_{i\,t}$$

**TABLE 1:** Table of research variables.

Variable properties	Variable identification	Measurement content	Measuring method
Explained variable	*RiskT*	Risk bearing level	Values calculated according to models (1)-(2).
Explanatory variable	*ICW*	Internal-control willingness	Virtual variable: two classification measurement based on Python machine learning “prediction” results; Positive willingness of internal control, recorded as 1; other wishes are 0.
Explained variable and comparison variable (*IC*)	*ICE*	Internal-control effectiveness	Dummy variable: effective internal control (and the conclusion was not denied in the follow-up audit) is 1; others (internal control is invalid or not disclosed in time according to the requirements of laws and regulations) are 0.
	*ICA*	Internal-control audit conclusion	Dummy variable: the standard unqualified opinion is 1; others (unqualified opinions with emphasis, unable to express opinions, negative opinions or not disclosed in time) are 0.
Moderator	*State*	Enterprise nature	Dummy variable: the state-owned enterprise takes the value of 1, and the others are 0.
Control variable	*Size*	company size	Natural logarithm of average total assets
	*Lev*	financial leverage	Financial leverage = Total Liabilities / total assets
	*BS*	Board size	Natural logarithm of the total number of directors
	*ROID*	Ratio of independent directors	Ratio of the number of independent directors to the total number of directors
	*EI*	Executive Incentive	Sum of shareholding ratio of senior executives (Chairman, CEO, CFO, and Chairman of the Board of Supervisors)
	*Growth*	Growth index	Expressed by the year-on-year growth rate of total operating revenue
	*FAge*	Years of establishment of the company	Natural logarithm of the company’s establishment time (years + 1)
	*Herf*	Ownership concentration	Select Herfindahl 10 index, which is the square of the shareholding proportion of the top 10 shareholders.
	*Ind*	Industry	Primary industry classification according to the standards of CSRC
	*Year*	Particular year	Year value

Among them, the explained variable *IC* represents one of the enterprise’s internal-control level *ICE* or *ICA*; the explained variable *RiskT* represents the level of enterprise risk-taking; the explanatory variable *ICW* represents the internal-control willingness.

### Research samples and data sources

We take the listed companies on China’s A-share main board as the research sample and select the information disclosed in the internal-control self-assessment report, annual financial report, and audit report of the sample enterprises from 2011 to 2018 as the data source. The selection rules for sample intervals are as follows: (1) According to the “Notice on Printing and Distributing Supporting Guidelines for Enterprise Internal Control” issued by the Ministry of Finance of China in 2010, since 1 January 2012, companies listed on the main boards of the Shanghai Stock Exchange and Shenzhen Stock Exchange will implement the “Enterprise Internal-control Evaluation Guidelines” and “Corporate Internal-control Audit Guidelines.” Additionally, it will be implemented for companies that are listed both domestically and overseas. It can be seen from this that the information disclosure of internal-control evaluation and audit reports of A-share main board listed companies mainly starts from the annual report time of 2011. We take the 2011 annual report as the starting point for the selection interval of the sample data. (2) In 2019, China and the world discovered the COVID-19, which seriously affected corporate operating data, information disclosure, and market performance. To achieve the comparability of corporate data, we take the 2018 annual report as the end point of the sample data selection interval.

At the same time, the sample enterprises are selected to sort out the primary samples according to the following rules: (1) The samples of finance and insurance industries are excluded because the financial statements of these listed companies have a special structure; and (2) samples with incomplete data are eliminated because the comparability of data samples cannot be guaranteed. The data sources of this paper include the followings: Wind, CSMAR database, DIB Internal Control and Risk Management database, CNINFO, and the website of Shanghai and Shenzhen Stock Exchange. To reduce the influence of extreme values, all continuous variables are winsorized according to 1 and 99% quantiles.

## Analysis of empirical results

### Descriptive statistics

Table 2 reports the descriptive statistics of the variables in this study. First, the mean *ICW* of the explanatory variable internal-control willingness is 0.19, indicating that from 2011 to 2018, 19% of the sample enterprises held positive subjective willingness of internal control. Second, among the explanatory variables, the average *RiskT* of enterprise risk-taking level is 0.09 and the median is 0.04; it shows that a small number of sample enterprises have a high level of risk-taking, which raises the average value of the overall *RiskT* of the sample. Third, the mean values of the explained variable and comparison variable *ICE* and *ICA* are 0.90 and 0.82, respectively, indicating that from 2011 to 2018, the internal-control level of more than 4/5 A-share main board listed companies is of high quality. The results reported in this table are condensed data, which are consistent with the data of regression analysis.

**TABLE 2:** Descriptive statistical results.

Stats	*N*	Mean	SD	Min	P10	P25	Median	P75	P90	Max
*RiskT*	9,236	0.09	0.21	0.01	0.01	0.02	0.04	0.07	0.13	1.61
*ICW*	9,236	0.19	0.36	0.00	0.00	0.00	0.00	0.00	1.00	1.00
*ICE*	9,236	0.90	0.28	0.00	1.00	1.00	1.00	1.00	1.00	1.00
*ICA*	9,236	0.82	0.38	0.00	0.00	1.00	1.00	1.00	1.00	1.00
*State*	9,236	0.66	0.38	0.00	0.00	0.00	1.00	1.00	1.00	1.00
*Size*	9,236	22.45	1.35	19.44	20.84	21.62	22.39	23.34	24.33	26.25
*Lev*	9,236	0.49	0.19	0.07	0.22	0.33	0.50	0.63	0.71	0.83
*BS*	9,236	2.20	0.21	1.60	1.93	2.07	2.21	2.20	2.41	2.73
*ROID*	9,236	0.37	0.05	0.33	0.33	0.34	0.35	0.40	0.43	0.57
*EI*	9,236	0.02	0.07	0.00	0.00	0.00	0.00	0.00	0.02	0.47
*Growth*	9,236	0.12	0.35	−0.61	−0.19	−0.05	0.08	0.21	0.42	2.21
*FAge*	9,236	2.99	0.25	2.08	2.70	2.82	3.00	3.17	3.29	3.58
*Herf*	9,236	0.34	0.19	0.04	0.12	0.20	0.31	0.46	0.59	0.83

### Probit regression test of internal-control willingness and enterprise internal-control level

Panel A in Table 3 reports the probit regression results between internal-control willingness and enterprise internal-control level. The results of column (1)-(2) show that *ICW* is positively correlated with *ICE* and *ICA* at the 1% significance level; it shows that the subjective initiative factor of internal control, that is, the willingness of internal control, can significantly and positively affect the final result of the level of internal control, that is, the enterprise holds the willingness of positive internal control, the probability of “effective internal control” in the internal-control evaluation conclusion is higher, and the rate of “standard unqualified opinion” in the internal-control audit conclusion is higher.

**TABLE 3:** Regression results of test model.

Panel A	(1)	(2)	(3)	(4)	Panel B	(5)	(6)	(7)
Variables	*ICE*	*ICA*	*ICE*	*ICA*	Variables	*RiskT*	*RiskT*	*RiskT*
*ICW*	1.15***	0.95***	0.68***	0.47***	*ICW*	−0.01***	−0.01***	−0.01**
	(12.75)	(10.87)	(4.82)	(4.45)		(−3.16)	(−2.78)	(−2.27)
*State*	NO	NO	NO	NO	*State*	NO	NO	−0.00*
	NO	NO	NO	NO		NO	NO	(−1.77)
ICW* State	NO	NO	NO	NO	ICW* State	NO	NO	−0.00**
	NO	NO	NO	NO		NO	NO	(−2.32)
*Size*	0.29***	0.25***	NO	NO	*Size*	−0.01***	NO	−0.01***
	(11.87)	(11.32)	NO	NO		(−9.45)	NO	(−9.20)
*Lev*	−0.01***	−0.01***	NO	NO	*Herf*	0.07***	NO	0.07***
	(−2.75)	(−4.68)	NO	NO		(9.01)	NO	(8.89)
*BS*	0.52***	1.13***	NO	NO	*Growth*	0.01***	NO	0.01**
	(2.99)	(2.71)	NO	NO		(2.89)	NO	(2.39)
*ROID*	1.24**	0.58**	NO	NO	*FAge*	−0.01***	NO	−0.01***
	(2.43)	(2.03)	NO	NO		(−3.02)	NO	(−2.93)
*EI*	−0.76***	−0.33**	NO	NO	*EI*	0.03**	NO	0.03**
	(−2.95)	(−2.11)	NO	NO		(2.52)	NO	(2.35)
*Constant*	−8.78***	−7.01***	−4.27***	−3.76***	*Constant*	0.23***	0.06***	0.20***
	(−13.86)	(−12.79)	(−5.19)	(−4.93)		(6.97)	(3.77)	(6.51)
*Year* & *Ind*	YES	YES	YES	YES	*Year* & *Ind*	YES	YES	YES
Observations	9,236	9,236	9,236	9,236	Observations	9,236	9,236	9,236
Pseudo R^2^	0.44	0.35	0.27	0.20	Adj-R^2^	0.83	0.56	0.81
LR chi^2^	2093.41***	1738.51***	1282.50***	950.39***	F	871.22***	571.30***	859.65***

***, **, and * are significant at the level of 1, 5, and 10%, respectively (the same below).

The economic interpretation of the empirical results is that, when *ICW* changes by 1 standard deviation 0.36, *ICE* changes positively by about 0.41 (1.15 × 0.36) units, and *ICA* changes positively by about 0.34 (0.95 × 0.36) units. This shows that the impact of internal-control willingness on the internal-control effectiveness is greater than the impact on the internal-control audit conclusion.

Among the control variables, *Size* is positively correlated with *ICE* and *ICA* at 1% significance level, indicating that larger company size is conducive to the formation of a high level of internal control; *Lev* is negatively correlated with *ICE* and *ICA* at 1% significance level, respectively, indicating that excessive leverage may reduce the level of internal control; *BS* and *ROID* were positively correlated with *ICE* and *ICA* at the significance level of 1 or 5%, respectively; it shows that the perfection of senior management and corporate governance, including a larger board of directors and a higher proportion of independent directors, is conducive to the formation of a high level of internal control; *EI* was negatively correlated with *ICE* and *ICA* at the significance level of 1 or 5%; it shows that the incentive measures of excessively increasing shareholding proportion of senior executives reduce the level of internal control. In addition, the *Pseudo R^2^* of the model is 0.44 and 0.35, respectively; *LR chi^2^* are significant, which proves that the model is credible.

The results of column (3)-(4) are the regression results between *ICW* and *ICE*, or between *ICW* and *ICA*, without control variables, and are similar to the main test results. Hypothesis 1 is confirmed.

### Analysis on the impact of internal-control willingness on enterprise risk-taking level

Panel B in Table 3 reports the regression results between internal-control willingness (*ICW*) and enterprise risk-taking level (*RiskT*). The results of column (5) show that *ICW* is negatively correlated with *RiskT* at the 1% significance level. It shows that internal-control willingness has a significant negative impact on the level of enterprise risk-taking.

The economic interpretation of the empirical results is that, when *ICW* changes by 1 standard deviation 0.36, *RiskT* changes negatively by about 0.004 (0.01 × 0.36) units. This is in line with corporate practice and previous research experience of actively fulfilling and complying with the Internal-control Guidelines in China could significantly reduce the risk of financial crisis and distress and then lower corporate risk-taking level (Jin et al., 2013; Li et al., 2021).

Among the control variables, most of the control factors related to *RiskT* are basically consistent with the previous research conclusions, including *Size* is negatively correlated with *RiskT* at the 1% significance level, that is, the risk-taking level of large enterprises is low; *Herf* is positively correlated with *RiskT* at the 1% significance level, that is, enterprises with high equity concentration have a higher risk-taking level. Meanwhile, we find that *Growth* is positively correlated with *RiskT* at the 1% significance level, indicating that fast-growing enterprises have a higher level of risk-taking; *FAge* is negatively correlated with *RiskT* at the 1% significance level, that is, enterprises with long-term establishment may have a low level of risk-taking; *EI* is positively correlated with *RiskT* at the 5% significance level, that is, equity incentive increases the enterprise risk-taking level.

The results of column (6) are the regression results between *ICW* and *RiskT* without control variables and are similar to the main test results. Thus, based on the empirical data results above, we believe that internal-control willingness can lower corporate risk-taking level. Hypothesis 2 is confirmed.

The mechanism by which internal-control willingness has a negative impact on corporate risk-taking is as follows: internal-control willingness is the willingness of constructing and executing internal-control system, which is one of the positive constituent factors of high quality internal-control level (*ICE* or *ICA*). At the same time, previous studies have shown that high-quality internal-control level had a significantly negative impact on corporate risk-taking level (Bargeron et al., 2010; Baugh et al., 2021). Therefore, from logical and empirical evidences, internal-control willingness has a negative impact on corporate risk-taking.

### Further research: Moderating effects of enterprise nature

In China, the nature of property rights is a unique phenomenon. Studies have shown that the nature of property rights can significantly affect the internal control of enterprises and their economic consequences (Zhang et al., 2013). Therefore, logically, state-owned enterprises (SOEs) may be heterogeneous.

Column (7) of Table 3 reports the regression results between *ICW*, *State*, and *RiskT* after introducing the property rights to form the interaction term. The results show that, after introducing the moderating factor of property rights, the interaction term between *ICW* and *State* is negatively correlated with *RiskT* at the 5% significance level. The results show that, state-owned enterprises to strengthen internal-control willingness, their risk-taking level is significantly lower than that of non-state-owned enterprises. This highlights the heterogeneity in the enterprise nature of different firms.

We can find some clues from descriptive statistics about the heterogeneity of property rights. According to the statistical results in Table 2, 66% (*State*) of the main board listed companies are state-owned enterprises. This is significantly different from the nature of enterprises in western capital markets where private enterprises are the main shareholders. The “post-holding characteristics” of state-owned enterprise leaders may be the main reason (Zhang et al., 2013). Because this inference is beyond the scope of empirical results, we will elaborate on it in the “Discussion” section.

### Robustness check

#### Balance panel data verification

Due to the limitations of many data sources such as internal-control regulations and database data sources, the above research adopts the method of pooled data. To enhance robustness and eliminate the influence of sample survival selection and missing variables, this paper converts the above-mentioned mixed data set into a balance panel, that is, to maintain the individual balance of each cross-sectional observation sample from 2011 to 2018; however, 3,876 observations were lost, and a new sample set containing 5,360 observations was obtained.

Panel A in Table 4 reports the regression results of fixed effects model (FE) after the conversion of mixed data set to balanced panel data set. The results showed that the correlation direction and significance of *ICW* and *RiskT* were consistent, and the significance level decreased from 1 to 5%. The results above also show the advantages of using pooled data as the main test data processing in this study.

**TABLE 4:** Robustness test results.

Panel A	(1)	Panel B	(2)	(3)	Panel C	(4)
Variables	*RiskT*	Variables	*ICE*	*ICA*	Variables	*RiskT*
*ICW*	−0.01**	*ICW*	1.05***	0.72***	*ICW*	−0.01***
	(−2.49)		(9.26)	(7.96)		(−2.84)
*Size*	−0.00***	*Size*	0.27***	0.26***	*Size*	−0.01***
	(−4.78)		(6.15)	(6.02)		(−5.02)
*Herf*	0.05***	*Lev*	−0.01**	−0.01***	*Herf*	0.06***
	(3.68)		(−2.43)	(−2.71)		(5.16)
*Growth*	0.00*	*BS*	0.46***	1.02**	*Growth*	0.00**
	(1.88)		(2.71)	(2.44)		(2.05)
*FAge*	−0.01**	*ROID*	0.75**	0.32*	*FAge*	−0.01***
	(−2.43)		(1.99)	(1.68)		(−2.71)
*EI*	0.02*	*EI*	−0.70**	−0.29*	*EI*	0.03**
	(1.71)		(−2.10)	(−1.82)		(2.40)
*Constant*	0.19***	*Constant*	−7.69***	−6.30***	*Constant*	0.18***
	(3.88)		(−7.14)	(−6.85)		(4.53)
*Year* & *Ind*	YES	*Year* & *Ind*	YES	YES	*Year* & *Ind*	YES
Observations	5,360	Observations	3,576	3,576	Observations	3,576
*R*^2^(between)	0.76	Pseudo R^2^	0.40	0.32	Adj-R^2^	0.72
Wald Chi^2^/F	2520.39***	LR chi^2^	558.92***	470.11***	Wald Chi^2^/F	658.52***

#### Propensity score matching test

The main test of this paper uses the measurement method based on Python text analysis and machine learning as the measurement method of internal-control willingness, due to the small proportion of internal-control “positive willingness” of listed companies on the A-share main board, accounting for only about 19% of all samples. Therefore, we use the propensity score matching method (PSM) to test the robustness of the model under the condition of approximate matching. Specifically, we take the corresponding control factors confirmed in the main test equation as the matching conditions and set the matching ratio as 1:1. The new sample sets of 3,576 samples are obtained for the clear outcome variable *ICE* and *ICA*, respectively, and the new sets of 3,576 samples are also obtained for the clear outcome variable *RiskT*. Then, we retest the main test.

Panel B in Table 4 reports probit regression results after PSM matching with *ICE* and *ICA* as clear outcome variables. Among them, the results of column (2)-(3) show that *ICW* was positively correlated with *ICE* and *ICA* at 1% significance level.

Panel C in Table 4 reports OLS regression results after PSM matching with *RiskT*. The results of column (4) show that *ICW* and *RiskT* are still negatively correlated at the significance level at 1%. In addition, the direction and significance of each control variable are basically consistent with the original main test, and the Adj-R^2^, LR chi^2^, and *F* values are similar, which proves that the original test result is robust and eliminates the endogenous doubts of this kind.

## Discussion

According to Positive Accounting Theory, the growing risk of bankruptcy is associated with the phenomenon of earning management (Chen et al., 2020; Durana et al., 2021). Earning management is one of the behaviors that is subjectively divorced from the internal control of an enterprise. Additionally, risk of bankruptcy is an extreme form of corporate risk-taking. Therefore, from the previous research evidence and academic conclusions, there is an inevitable relationship between internal-control willingness and the level of enterprise risk-taking. Moreover, in enterprise practice, the internal-control defects of Enron, Societe Generale, and China Guitang all expose the phenomenon of subjective manipulation, resulting in increased business risks, abnormal stock prices, huge economic losses, or bankruptcy (Hudakova et al., 2021; Oulehlova et al., 2021).

At the same time, the nature of property rights is a unique phenomenon in China. The heads of state-owned enterprises have the “post-holding characteristics” and pay attention to external evaluations and their own progress (Zhang et al., 2013). Meanwhile, the behavior of state-owned enterprises to strengthen internal-control willingness will help the outside world to recognize the internal-control work of the heads of state-owned enterprise and help to optimize the promotion performance of enterprise leaders. Therefore, state-owned enterprises to strengthen internal-control willingness, their risk-taking level is significantly lower than that of non-state-owned enterprises.

## Research conclusions, implications, and limitations

### Research conclusions

This paper puts forward the subjective factors of the construction and implementation of internal control, that is, the internal-control willingness, and its confirmation and measurement methods; moreover, through the internal-control and financial data of Chinese A-share main board enterprises from 2011 to 2018, this paper tests the possible impact of internal-control willingness on the level of enterprise risk-taking. The conclusions are as follows:

(1) The internal-control willingness has a positive impact on the level of internal control. The specific performance is as follows: the enterprise holds a positive willingness of internal control, the probability of “effective internal control” in the internal-control evaluation conclusion is higher, and the rate of “standard unqualified opinion” in the internal-control audit conclusion is higher.

(2) The internal-control willingness helps to lower corporate risk-taking level. The mechanism includes the following: internal-control willingness is the willingness of constructing and executing internal-control system, which is one of the positive constituent factors of high-quality internal-control level. At the same time, previous studies have shown that high-quality internal-control level had a significantly negative impact on corporate risk-taking level (Bargeron et al., 2010; Baugh et al., 2021). Therefore, from logical and empirical evidence, internal-control willingness has a negative impact on corporate risk-taking.
(3) Further research finds that state-owned enterprises strengthen internal-control willingness and their risk-taking level is significantly lower than that of non-state-owned enterprises.

### Research implications

The implications of this paper lie in as follows: on the one hand, the subjective initiative factors are introduced into the research field of internal control, and it is found that the willingness of internal control is an important factor that affects the level of internal control. On the other hand, the subjective initiative factors verify the relevance and mechanism between internal-control willingness and enterprise risk-taking and provide decision-making basis for enterprise stable operation from the perspective of risk control.

The enlightenment of this research conclusion is as follows: the regulatory authorities should actively urge the enterprise board of directors to strengthen the cultivation of internal-control willingness, which is not only conducive to the construction and implementation of enterprise internal-control system, but also conducive to the decline of enterprise risk-taking level.

### Research limitations

There are some limitations in this paper: First, for the measurement of corporate internal-control willingness, we use measurement methods based on text analysis and machine learning, and other measurement methods still need to be explored in follow-up researches. Second, the impact of internal-control willingness on enterprise risk management is differentiated. In this paper, we only discuss its impact on the overall risk-taking level, and the inhibitory effect on specific risks of enterprises needs to be discovered in subsequent researches.

## Data availability statement

The raw data supporting the conclusions of this article will be made available by the authors, without undue reservation.

## Author contributions

LC: acquisition of the data and analysis, interpretation of the data, and drafting the manuscript. YL: design of the study. BL: conception of internal-control willingness, providing a measurement method, and correspondence. All authors contributed to the article and approved the submitted version.

## Conflict of interest

The authors declare that the research was conducted in the absence of any commercial or financial relationships that could be construed as a potential conflict of interest.

## Publisher’s note

All claims expressed in this article are solely those of the authors and do not necessarily represent those of their affiliated organizations, or those of the publisher, the editors and the reviewers. Any product that may be evaluated in this article, or claim that may be made by its manufacturer, is not guaranteed or endorsed by the publisher.
